# Fe-phthalocyanine derived highly conjugated 2D covalent organic framework as superior electrocatalyst for oxygen reduction reaction

**DOI:** 10.1186/s11671-023-03890-w

**Published:** 2023-09-04

**Authors:** Anuj Kumar, Mohd Ubaidullah, Bidhan Pandit, Ghulam Yasin, Ram K. Gupta, Guoxin Zhang

**Affiliations:** 1 Nano-Technology Research Laboratory, Department of Chemistry, GLA University, Mathura, Uttar Pradesh 281406 India; 2 Department of Chemistry, College of Science, King Saud University, 11451 Riyadh, Saudi Arabia; 3 Institute for Advanced Study, Shenzhen University, Shenzhen, 518060 Guangdong China; 4 Department of Chemistry, National Institute for Materials Advancement, Pittsburg State University, Pittsburg, KS 66762 USA; 5 Department of Electrical Engineering and Automation, Shandong University of Science and Technology, Qingdao, 266590 Shandong People’s Republic of China; 6 Department of Materials Science and Engineering and Chemical Engineering, Universidad Carlos III de Madrid, Avenida de La Universidad 30, 28911 Leganés, Madrid, Spain

**Keywords:** Molecular catalysts, Fe-phthalocyanine, Oxygen reduction reaction, Electrocatalytic activity

## Abstract

Although porphyry systems like metallo-phthalocynine are recognized as promising molecular models for electrocatalytic oxygen reduction reaction (ORR), their poor durability and methanol tolerance are still challenges and need improvement before being considered for practical applications. Herein, we successfully designed and constructed a Fe-phthalocyanine-derived highly conjugated 2D covalent organic framework (2D FePc-COF), using octa-amino-Fe-phthalocyanine (OA-FePc) and cyclohexanone as precursors. The prepared 2D FePc-COF was characterized via multiple analytic techniques. The electrochemical studies indicated that prepared 2D FePc-COF was far more superior to OA-FePc and 20% Pt/C, displaying anodic shift of 100 and 50 mV (vs RHE) in formal potential, respectively. Moreover, this catalyst also demonstrated excellent methanol tolerance and durability (over 10,000 CV cycles). Theoretical investigations revealed that due to extended conjugation and elimination of electron donating groups (-NH_2_), the shifting of dz^2^-orbital (Fe) energy took nearer to π*-orbital (O_2_), allowing optimum coupling of both the orbitals, thereby enhancing 4e^−^ ORR. This work demonstrates the art of molecular design, aiming at improving catalytic activity of macrocyclic molecular systems towards ORR.

**Supplementary Information:**

The online version contains supplementary material available at 10.1186/s11671-023-03890-w.

## Introduction

The electrocatalytic oxygen reduction reaction (ORR) is one of the important processes for the development of the renewable energy sector [1–4]. However, due to high O–O bond reorganization energy, this process demonstrates extremely sluggish kinetics at the cathode in fuel cells, requiring efficient electrocatalysts [5, 6]. Although Pt-containing catalysts can enhance the ORR kinetics, their limited activity and durability, as well as high cost, hinder their practical application, leaving the scientific community with the challenge of developing desirable, optimized electrocatalysts in the area [7, 8]. Responding to the task, extensive research has been carried out to fabricate useful heterogeneous catalysts including transition metal oxides [9], sulfides [10], phosphides [11], and atomically dispersed single/dual-atom catalysts [12, 13], which are easier to develop having good activity and remarkable stability with the limitation of difficulties in chemical modification to improve electrocatalytic activity [14–16].

Porphyry systems, on the other hand (particularly metallo-phthalocyanines, MPcs), are good molecular models for ORR because they can be tailored through framework substitution as well as the replacement of the central metal atom [17]. Out of all, substitution appears to be the best option to alter their ORR performance, and it has been observed that electron withdrawing or donating groups (EWGs and EDGs) decreased or increased the electron density around the active site (MN_4_-moieties), enhancing or inhibiting their ORR activity, respectively [18]. Usually, EDGs on metallo-phthalocyanines can raise dz^2^-orbital energy of a central metal atom, shifting dz^2^-orbital closer to antibonding pi-orbital (π*) of O_2_, thus favor facilitation of e- transfer from dz^2^-orbital to π*-orbital, weakening the O–O bond, thereby, improving their ORR activity and vice versa for EDGs on MPc catalysts. Although such implementations have improved the ORR activity of MPc catalysts, their durability during ORR is still poor [19]. Therefore, the concerned scientific community tried to incorporate MPc in large conjugated frameworks including metal–organic frameworks (MOF) as well as covalent organic frameworks (COFs) to improve their durability during ORR [20]. In the presence of electrolytes, these catalysts are found to be stable, however, a great challenge still exists to optimize their activity via a structural change at the molecular level. COFs are the crystalline porous framework and can be tuned for ORR improvement [21], the reason being the ability of such material to work utilizing the added benefits of molecular as well as heterogeneous catalysts [22]. These materials offer the scope of accurate manipulation of the spatial arrangement of catalytic centers insight into the pre-decided COF's structure by virtue of constructing with molecular building units [23–25], and the possibility of constructing with molecular building units. The additional advantage of these frameworks is their capability to carry out multi-variate synthesis while maintaining identical topology and the possibility of incorporating functionally modified building units into the structure [26]. This method can produce a material possessing emergent characteristics more than the combined molecular parts [27, 28]. Considering such ideas, several FePc-based MOFs and COFs have been designed and fabricated for ORR, however, their performance was not as expected, which might be due to a lack of optimized energy levels and extended free electronic conjugation [29, 30].

In this work, we utilized octa-amino-Fe-phthalocyanine (OA-FePc), a poor ORR active molecular model due to EDGs on Pc framework and cyclohexanone as building blocks to construct a FePc-based COF (2D FePc-COF). The created 2D FePc-COF displayed superior ORR activity than OA-FePc and a traditional catalyst, such as 20% Pt/C. In addition, the 2D FePc-COF catalyst exhibited exceptional durability as well as methanol tolerance, suggesting its capability at a practical level. Density functional theory suggested possible migration of dz^2^-orbital (Fe) energy near to π*-orbital (dioxygen) due to prolonged conjugation and elimination of -NH_2_ (EDGs), allowing optimal coupling of both the orbitals and boosting 4e^−^ ORR. The novelty of this work is that (i) it offers the opportunity of improving ORR activity of EDGs substituted FePc, (iii) it provides the maximum ORR performance (activity, durability, and methanol tolerance) for 2D FePc-COF, which has never been achieved before for FePc-based materials, and (ii) it offers a facile single-step microwave-assisted strategy to prepare 2D COFs using ketonic and amino-based building blocks. Moreover, the 2D-FePc-COF catalyst was prepared using inexpensive raw precursors, and the total cost of the constructed catalyst is significantly lower than that of the noble metal Pt catalyst. Therefore, 2D-FePc-COF catalyst can be used as cathode material in fuel cells, contributing to the low-cost development of fuel cell technology. In this work, we used microwave heating to approach the desired chemical change between the ketonic and amino-based building blocks because microwave energy offers a uniform heating environment.

## Experimental section

### Preparation of 2D FePc-COF

To prepare 2D FePc-COF, one mole of cyclohexanone and three moles of OA-FePc were added to a mixed solvent containing 30 mL of each xylene and ethanol. To achieve homogeneity, it was then subjected to 2 h of sonication at 45 °C. The obtained reaction mixture was transferred to a microwave reactor, and the reaction condition was adjusted to 300 W and a 5 °C/min reaction rate to achieve 120 °C. The reaction was then continued for 2 h. The resulting dark green sticky material was concentrated in a rotatory evaporator at 70 °C. The solid content was then washed with a solution consisting of equimolar acetone and ether followed by washing ethanol and dried at 100 °C overnight [24].

## Results and discussion

### Physiochemical characterization of 2D FePc-COF

**Fig. 1:**
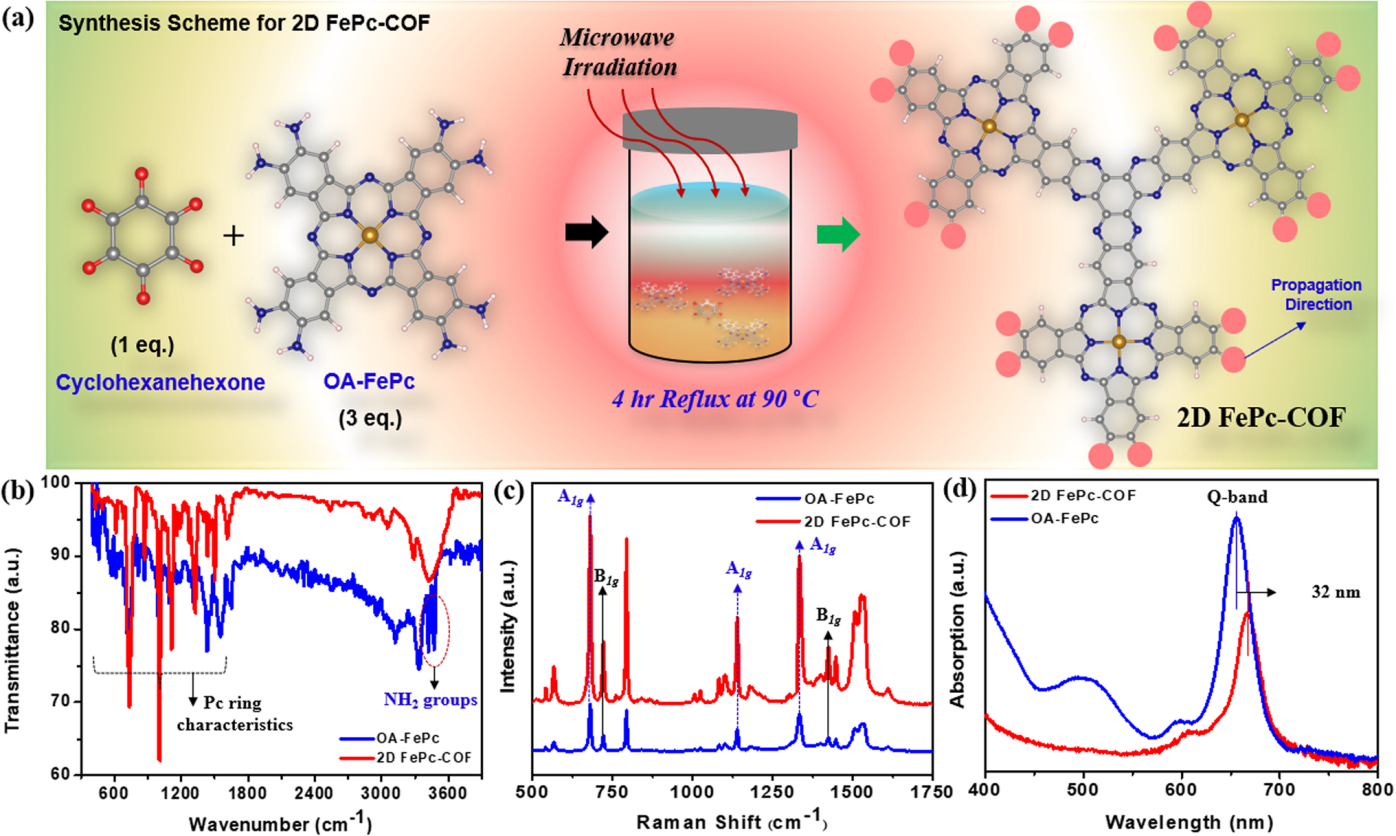
**a** Illustration of preparation approach for 2D FePc-COF. Spectra recorded with **b** FTIR, **c** Raman, and **d** UV–Vis spectroscopic techniques for OA-FePc and 2D FePc-COF electrocatalysts

In the present work, a reaction between cyclohexanone and OA-FePc was approached under microwave-assisted condensation to prepare the 2D FePc-COF, as shown in Fig. 1a. This method is preferable since this reaction involves only one step and produces a high yield of the product. Further, to verify the functional groups in the structure of the synthesized 2D FePc-COF, FTIR spectra of both the 2D FePc-COF and OA-FePc were compared **(**Fig. 1b**)**. The results showed the disappearance of ketonic (due to cyclohexanone) and amino (due to OA-FePc) groups signals in the FTIR spectra of 2D FePc-COF, confirming the successful formation of 2D FePc-COF. Moreover, the C–H and C=N vibrational peaks (765–1470 and 1585–1650 cm^−1^) in 2D FePc-COF were found to be much stronger than in OA-FePc, which may be attributed to the extended conjugation and existence of a number of FePc moieties in the 2D FePc-COF sample [31]. The breathing and deformation of the C=C bond in the molecular frameworks of OA-FePc and 2D FePc-COF were seen as bands at 685 cm^−1^ and 776 cm^−1^, respectively, in their Raman spectra [32]. Furthermore, the bands close to 1570 cm^−1^ can be explained by stretching C-N–C bridge bonds **(**Fig. 1c**)**. On the other hand, the 2D FePc-COF signal had extraordinarily high peak intensities as well, which can be explained by the fact that 2D FePc-COF involves a longer conjugation [33].

Next, to investigate the electronic structure in the 2D FePc-COF, optical studies were conducted in methanol for both the OA-FePc and 2D FePc-COF systems. The Q-band, which is defined as the e- transition from HOMO to LUMO, appeared in the range of 645–670 nm for both the samples **(**Fig. 1d**)** [33]. The Q-band of 2D FePc-COF shifted to a longer wavelength (32 nm) as compared to precursor OA-FePc, confirming the extension of conjugation due to the assembly of OA-FePc and cyclohexanone. Longer conjugation in 2D FePc-COF is assumed to be responsible for the red-shift in Q-band, which is interpreted as a decrease in e_g_-orbital energy [31].

**Fig. 2:**
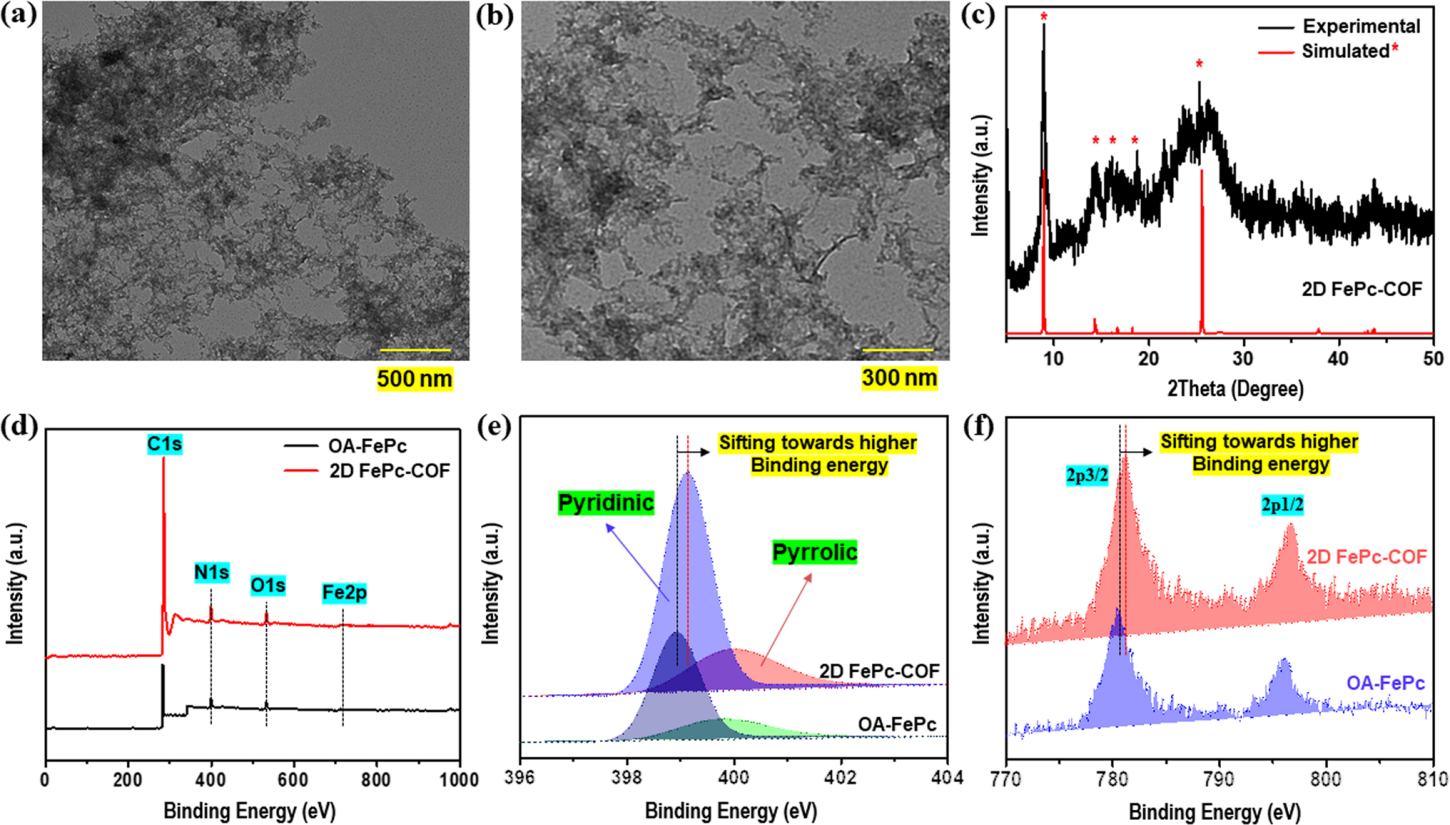
**a**–**b** TEM images for 2D FePc COF. **c** Experimental and simulated XRD pattern for 2D FePc COF. **d** XPS Survey for OA-FePc, and 2D FePc-COF. XPS scan for **e** N2p, and **f** Fe2p, in the 2D FePc COF sample

The TEM technique was utilized to carry out the morphological evaluation of the 2D FePc-COF sample. The TEM pictures (Fig. 2a–b) of 2D FePc-COF revealed its holey-sheet type morphology. Figure 2c showed both the experimental and simulated XRD patterns of the 2D FePc-COF, providing evidence of its successful formation. Nevertheless, the 2D FePc-COF exhibited an amorphous phase, as evidenced by its broad XRD signals [34]. In addition, XPS analysis was used to investigate the states and bonding of nitrogen and iron in the 2D FePc-COF sample. The XPS survey of OA-FePc and 2D FePc-COF is depicted in Fig. 2d, which indicates the existence of nitrogen and iron in both the catalysts. However, the projecting O1s evidence in XPS survey of 2D FePc-COF might be because of the O_ads_ on both the OA-FePc and 2D FePc-COF catalyst. Since FeN_4_ active sites are extremely responsive towards dioxygen, therefore, it got adsorbed on both the OA-FePc and 2D FePc-COF on air exposure. When compared to precursor OA-FePc, the N1s XPS spectra of 2D FePc-COF **(**Fig. 2e**)** showed a large positive shift in C-N/C=N binding energy. This indicated that there was a considerable change in the conjugation following the formation of COF. For 2D FePc COF, the envolved binding energy for Fe2p signal **(**Fig. 2f**)**, on the other hand, is relatively comparable with the precursor OA-FePc. It is possible to attribute the Fe2p signals to the 2p_3/2_ orbital of the Fe^2+^ state as well as the 2p_1/2_ orbital of the Fe^2+^ state [35, 36].

### Electrocatalytic ORR studies of 2D FePc-COF

**Fig. 3:**
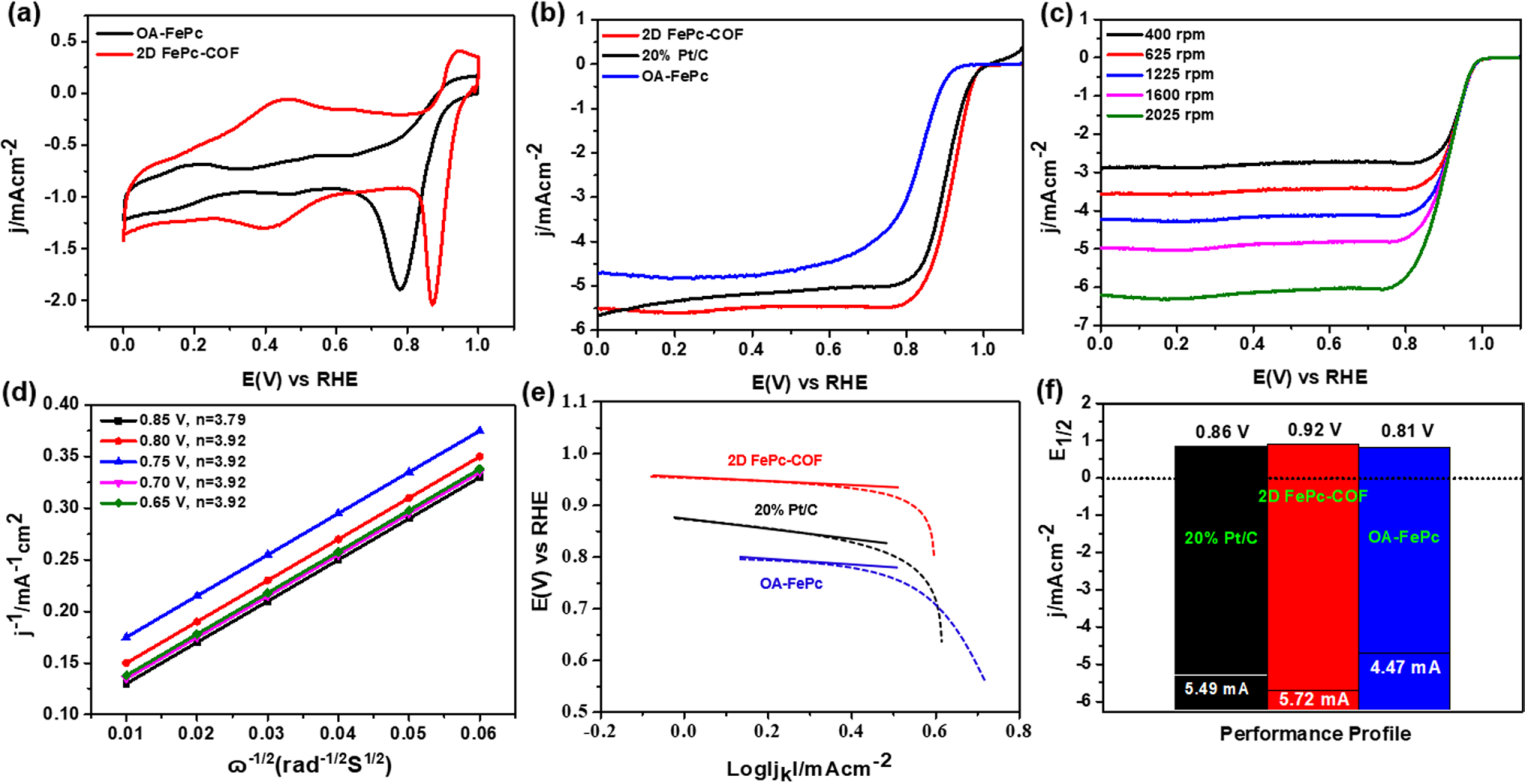
**a** Cyclic voltammograms, and **b** ORR polarization curves, for OA-FePc, 2D FePc COF, and 20% Pt/C catalysts, **c** ORR polarization curves recorded at different electrode rotation rates, and **d** K-L plots for for 2D FePc COF. **e** Tafel slopes for 20% Pt/C, OA-FePc and the 2D FePc COF. **f** A comparison between current density and *E*_1/2_ for OA-FePc, 2D FePc COF, and 20% Pt/C catalysts

To assess the ORR activity of OA-FePc precursor and prepared 2D FePc-COF catalyst was made by comparing the activity with commercial 20% Pt/C, and all the electrochemical tests (CV and RDE) were carried out in O_2_-soaked solution of 0.1 M KOH (*please see supporting information*). Cyclic and linear sweap voltammograms for these catalysts were obtained at 50 and 5 mV/s scan rates, respectively. Figure 3a showed the CV curve for OA-FePc and 2D FePc-COF catalysts, displaying the half-wave (*E*_1/2_) and onset potential of 0.74 V, 0.92 V, and 0.82 V, 0.97 V, respectively. ORR polarization curves for OA-FePc, 2D FePc-COF, and 20% Pt/C catalysts are shown in Fig. 3b, demonstrating an onset potential of 0.82 V, 0.97 V, and 0.96 V, and *E*_1/2_ of 0.73 V, 0.92 V, and 0.86 V, respectively [20]. The results of CV and polarization curves in terms of *E*_1/2_ and onset potential are very close, indicating precise measurements of both the techniques for OA-FePc and 2D FePc-COF catalysts. 2D FePc-COF catalyst demonstrated a 50 mV and 190 mV positive shift in *E*_1/2_ as compared to commercial 20% Pt/C and OA-FePc precursor, respectively, revealing superior ORR performance of 2D FePc-COF catalyst [30]. Moreover, the fact that the 2D-FePc COF catalyst had a high anodic shift in *E*_1/2_^ORR^ suggests its utility in getting a high electrochemical conversion efficiency in fuel cells at a practical level.

Further, to calculate the quantitative involvement of electrons in the ORR process, LSV curves were recorded in the range of 400–2025 rpm of electrode rotation speed **(**Fig. 3c**)**. The linearity of Kautecky-Levich plots suggested the first-order ORR electrode kinetics of 2D FePc-COF modified electrode at various potentials **(**Fig. 3d**)** [37]. Further, the slope of the 1/J curve and the ɷ^1/2^ curve indicated the involvement of approximately four electrons in the ORR process at the 2D FePc-COF modified electrode. Further, the obtained data of potential and current density for each sample was utilized to construct the corresponding Tafel slopes. The lower value of the Tafel slope for 2D FePc-COF (80 mV/dec) than OA-FePc (156 mV/dec) and 20% Pt/C (128 mV/dec) clearly suggested the improved ORR kinetics for 2D FePc-COF catalyst **(**Fig. 3e**)**. Therefore, lower onset potential of + 0.97 V, involvement of 3.93 electrons in ORR process and minimum Tafel slop for 2D FePc-COF catalyst demonstrated its superior ORR catalytic activity than commercial 20% Pt/C catalyst and OA-FePc **(**Fig. 3f**)** [38].

**Fig. 4:**
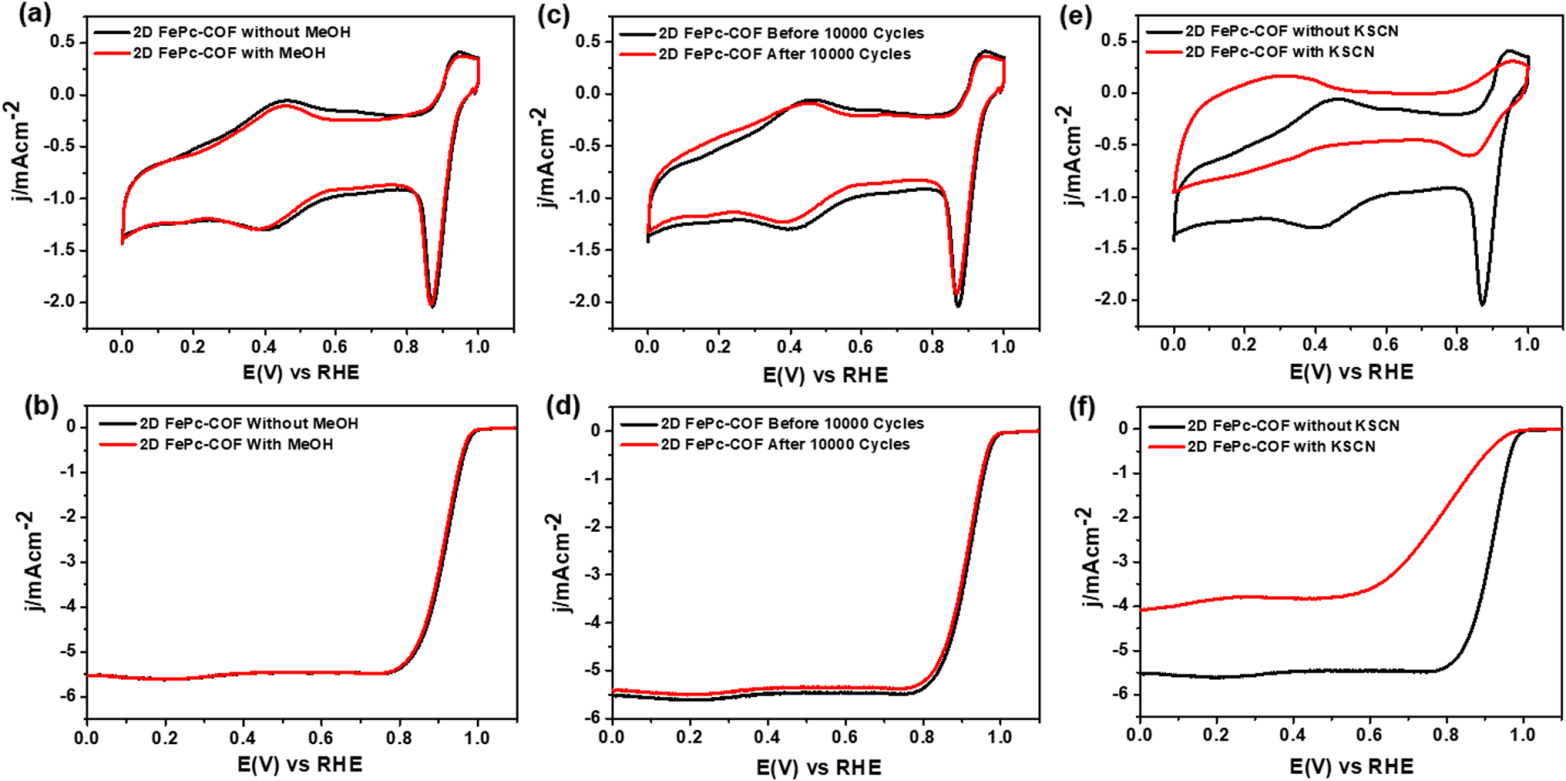
The **a** CV, and **b** LSV curves for 2D FePc-COF catalyst, recorded in O_2_-saturated KOH in presence and in absence of 5 mL MeOH. The **c** CV, and **d** LSV curves for 2D FePc-COF catalyst, recorded in O_2_-saturated KOH before and after 10,000 CV cycles. The **e** CV, and **f** LSV curves for 2D FePc-COF catalyst, recorded in O_2_-saturated KOH containing 1 mL KSCN

Besides, to assess the practical applicability of 2D FePc-COF in direct methanol fuel cells (DMFCs), its methanol tolerance was tested. The results suggested the exceptional tolerance of 2D FePc-COF against MeOH, demonstrating only a modest change in *E*_1/2_ (a loss of 5 mV) (Fig. 4a–b). Along with this property of 2D FePc-COF, its prospect as an ORR electrocatalyst at the industry level was assessed by LSV curves before and after performing 10,000 CV cycles in O_2_-soaked 0.1 M KOH. The LSV curve after 10,000 CV cycles for 2D FePc-COF displayed insignificant loss in *E*_1/2_ (3 mV) and current density (0.1 mA/cm^2^), demonstrating its exceptional durability during the ORR process (Fig. 4c–d) [39, 40]. Table 1 showed the comparison between prepared 2D FePc-COF catalysts with other reported catalysts towards ORR in literature.

**Table 1:** A comparison between prepared 2D-FePc COF catalyst with other reported catalysts towards ORR in literature

Electrocatalysts	Electrolyte	*E*_1/2_ (V)	E_onset_ (V)	J_L_ (mAcm^−2^)	References
20% Pt/C	0.1 M KOH	0.86 vs RHE	0.96 vs RHE	5.57	This work
OA-FePc	0.1 M KOH	0.74 vs RHE	0.82 vs RHE	4.61	This work
2D-FePc COF	0.1 M KOH	0.92 vs RHE	0.97 vs RHE	5.62	This work
Fe_2_Pc_2_	0.1 M KOH	− 0.14 *vs* SCE	− 0.04 *vs* SCE	5.53	[41]
FeAB–O	0.1 M KOH	+ 0.90 vs RHE	+ 0.95 vs RHE	6.10	[42]
FePc/(Fe-BP(N))	0.1 M KOH	+ 0.84 *vs* RHE	+ 0.95 vs RHE	6.12	[42]
Fe_2_Pc_2_	0.1 M KOH	− 0.16 *vs* SCE	− 0.06 *vs* SCE	4.95	[43]
FePc/GO	0.1 M KOH	− 0.32 *vs* SCE	–	–	[44]
FePc/GO/MWCNT	0.1 M KOH	–	− 0.07 *vs* SCE	–	[44]
FePc(CP)_4_/Gr	0.1 M KOH	− 0.23 *vs* SCE	− 0.069 *vs* SCE	–	[45]

Further, it is believed that for such FePc-based molecular catalysts, central FeN_4_ moieties act as active sites, where O_2_ adsorption on Fe and electron transfer from its dz^2^-orbital into π*-orbital of O_2_ take place, resulting in lengthening of O–O bond, which offers easy rupturing of O–O bond. In order to confirm whether or not FeN_4_ moieties acted as ORR active sites, the poisoning experiments with KSCN were performed on 2D FePc-COF. The CV of 2D FePc-COF after adding 1 mL of KSCN in O_2_-saturated KOH electrolyte showed a worsening in the ORR peak **(**Fig. 4e**)**, indicating FeN_4_-site blocking due to SCN-ion adsorption, as supported by the significant decrease in *E*_1/2_ of the LSV curve recorded in similar experimental conditions **(**Fig. 4f**)**.

### Theoretical study of 2D FePc-COF ORR catalyst

**Fig. 5:**
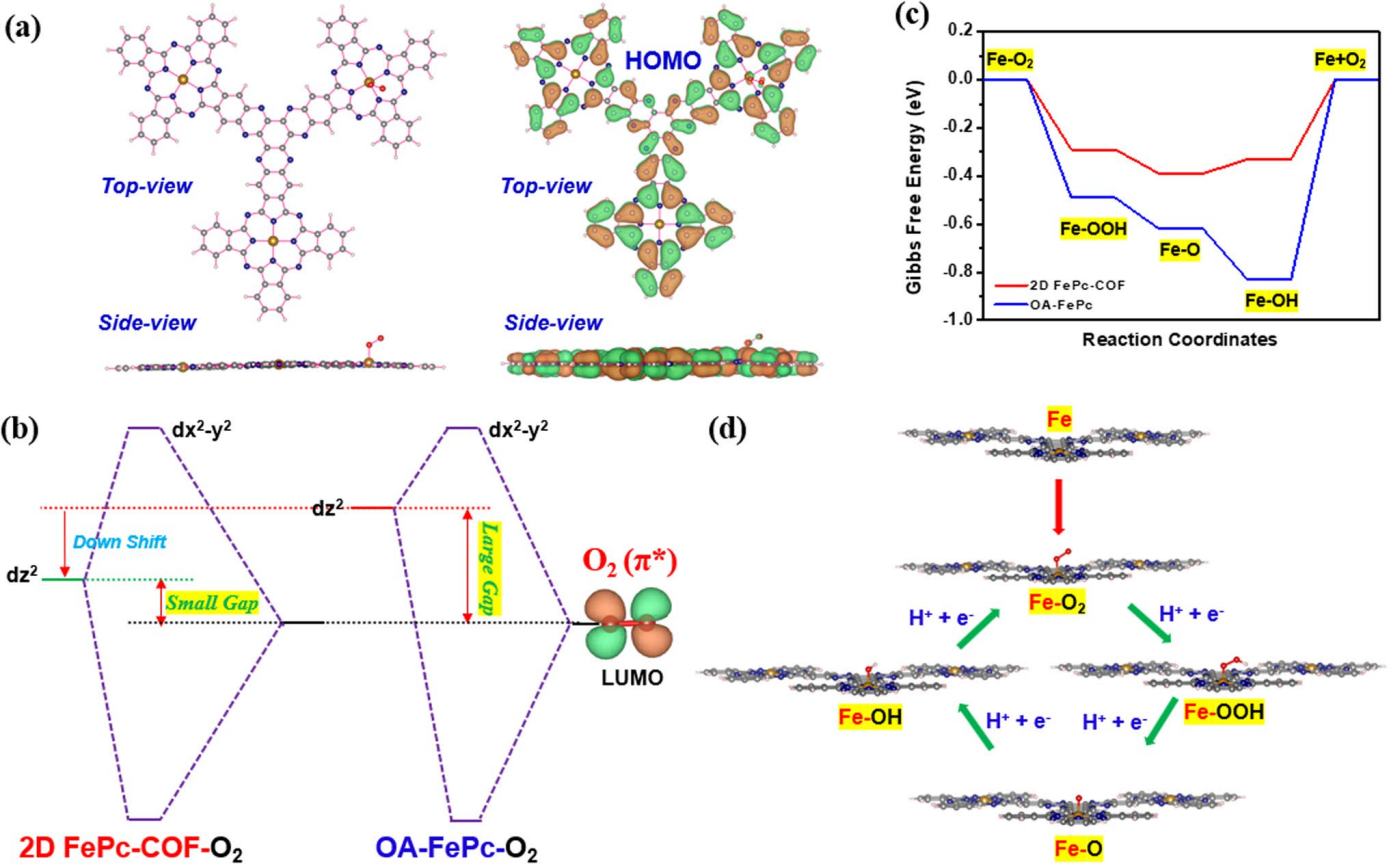
**a** Optimized geometry of 2D FePc-COF and HOMO–LUMO of 2D FePc-COF-O_2_ adduct, **b** representation of dz^2^ energy level of OA-FePc and 2D FePc-COF with respect to π*-orbital O_2_, **c** Free energy plots for OA-FePc and 2D FePc-COF catalysts, and **d** ORR mechanism on 2D FePc-COF

Further, the Gaussian 09 programme was exploited for the theoretical calculations to get deeper information about the superior ORR activity of the 2D FePc-COF electrocatalyst. The optimization results indicate that the average bond length of Fe and N in 2D FePc-COF (Fig. 5a) is shorter in comparison to OA-FePc (Fig. S1-S2). This suggests that following the condensation reaction between OA-FePc and cyclohexanone, the bonding between Fe and N becomes more stable due to an extended conjugation. Moreover, dz^2^ orbital energy was found to be lower for 2D FePc-COF as compared to OA-FePc (Fig. 5b) [37]. It can be attributed to high conjugation in 2D FePc COF catalyst, which reduced the dz^2^ orbital energy of Fe, as also verified by the longer shift in the UV–vis Q-band for 2D FePc-COF. The results of the optimization of dioxygen predicted that it would exist as a triplet; its O–O bond length was optimized to be 1.231, and its HOMO as well as LUMO energies (Fig. S3) were calculated as − 6.844 and − 4.561 eV, respectively. Frontier orbitals with similar energy, such as the dz^2^ orbital of Fe and π*-orbital of O_2_, are supposed to have optimum coupling, facilitating high charge transfer to π* orbital of O_2_ from the dz^2^ orbital of Fe-atom and weakening the O–O bond [38].

Figure 5b displays the down-shift nature of e_g_-orbital systematically that occurs as a result of large conjugation in 2D FePc-COF to create N_4_Fe-O_2_ adduct. In addition, optimization of all ORR intermediates, including OO*, OOH*, O*, and OH* on the FeN_4_ site of the 2D FePc-COF was performed. According to the findings, the fourth step of the ORR, which involved the disposal of *OH from the active sites of 2D FePc-COF, advanced with a 0.25 eV energy barrier than the energy barrier that existed for the precursor OA-FePc (0.84 eV) (Fig. 5c) [39]. The proposed ORR mechanism on 2D FePc-COF is shown in Fig. 5d. The results might explain why the ORR activity of 2D FePc-COF catalyst was much higher compared to that of the precursor OA-FePc, as also supported by increased O–O bond length in 2D FePc-COF-O_2_ adduct (Fig. S4-S5). The enhanced ORR activity of conjugated 2D FePc-COF catalyst is not only its initiation towards delocalization of electrons, in addition, but it also has a number of FeN_4_-active sites too, both of which in combination contribute to the overall performance of the 2D FePc-COF catalyst.

## Conclusion

In this investigation, the huge conjugated 2D FePc-COF was created by following a strategy that involved the use of a microwave, and it was then characterized by the spectroscopic, microscopic, and electrochemical methods. When compared with the precursor OA-FePc and commercial Pt/C electrocatalyst in O_2_-soaked KOH, the prepared 2D FePc-COF had improved ORR performance, demonstrating positive shifts in the formal potential of 100 and 50 mV, respectively. In addition, 2D FePc-COF displayed a low Tafel value (80 mV/dec) than Pt/C catalyst (128 mV/dec) and precursor octa-ammine-FePc (156 mV/dec), which is indicative of the quick ORR kinetics with the 2D FePc COF. Moreover, DFT calculations predicted extended conjugation in 2D FePc-COF would decrease dz^2^(Fe) orbital energy, moving it nearer to the π*-orbital (O_2_), allowing better coupling between orbitals, and leading to 4e-ORR with low overpotential. Moreover, 2D FePc-COF catalyst demonstrated a minimal tolerance for methanol and a high level of durability over 10,000 CV cycles. Therefore, by altering the energy of the active site, and incorporating FePc in a conjugated framework, this work opens the door to achieve the goal of boosting the performance of molecular and M–N-C catalytic systems.

### Supplementary Information

Additional file 1.

## Data Availability

The datasets generated during and/or analyzed during the current study are available from the corresponding author on reasonable request.

## References

[1] Martinez U, Babu SK, Holby EF, Chung HT, Yin X, Zelenay P (2019). Progress in the development of Fe-based PGM-free electrocatalysts for the oxygen reduction reaction. Adv Mater.

[2] Ibraheem S, Yasin G, Kumar A, Mushtaq MA, Ibrahim S, Iqbal R, Tabish M, Ali S, Saad A (2022). Iron-cation-coordinated cobalt-bridged-selenides nanorods for highly efficient photo/electrochemical water splitting. Appl Catal B.

[3] Yasin G, Ali S, Ibraheem S, Kumar A, Tabish M, Mushtaq MA, Ajmal S, Arif M, Khan MA, Saad A, Qiao L, Zhao W (2023). Simultaneously engineering the synergistic-effects and coordination-environment of dual-single-atomic iron/cobalt-sites as a bifunctional oxygen electrocatalyst for rechargeable zinc-air batteries. ACS Catal.

[4] Yasin G, Ibrahim S, Ibraheem S, Ali S, Iqbal R, Kumar A, Tabish M, Slimani Y, Nguyen TA, Xu H, Zhao W (2021). Defective/graphitic synergy in a heteroatom-interlinked-triggered metal-free electrocatalyst for high-performance rechargeable zinc–air batteries. J Mater Chem A.

[5] Wang J, Kong H, Zhang J, Hao Y, Shao Z, Ciucci F (2021). Carbon-based electrocatalysts for sustainable energy applications. Prog Mater Sci.

[6] Nadeem M, Yasin G, Arif M, Tabassum H, Bhatti MH, Mehmood M, Yunus U, Iqbal R, Nguyen TA, Slimani Y, Song H, Zhao W (2021). Highly active sites of Pt/Er dispersed N-doped hierarchical porous carbon for trifunctional electrocatalyst. Chem Eng J.

[7] Liu M, Zhao Z, Duan X, Huang Y (2019). Nanoscale structure design for high-performance Pt-based ORR catalysts. Adv Mater.

[8] Han A, Wang B, Kumar A, Qin Y, Jin J, Wang X, Yang C, Dong B, Jia Y, Liu J (2019). Recent advances for MOF-derived carbon-supported single-atom catalysts. Small Methods.

[9] Goswami C, Hazarika KK, Bharali P (2018). Transition metal oxide nanocatalysts for oxygen reduction reaction. Mater Sci Energy Technol.

[10] Cui M, Yang C, Li B, Dong Q, Wu M, Hwang S, Xie H, Wang X, Wang G, Hu L (2021). High-entropy metal sulfide nanoparticles promise high-performance oxygen evolution reaction. Adv Energy Mater.

[11] Lv Y, Wang X (2017). Nonprecious metal phosphides as catalysts for hydrogen evolution, oxygen reduction and evolution reactions. Catal Sci Technol.

[12] Liu M, Wang L, Zhao K, Shi S, Shao Q, Zhang L, Sun X, Zhao Y, Zhang J (2019). Atomically dispersed metal catalysts for the oxygen reduction reaction: synthesis, characterization, reaction mechanisms and electrochemical energy applications. Energy Environ Sci.

[13] Cheng Y, He S, Veder JP, Marco R, Yang SZ, Jiang SP (2019). Atomically dispersed bimetallic FeNi catalysts as highly efficient bifunctional catalysts for reversible oxygen evolution and oxygen reduction reactions. ChemElectroChem.

[14] Zhou Y, Abazari R, Chen J, Tahir M, Kumar A, Ikreedeegh RR, Rani E, Singh H, Kirillov AM (2022). Bimetallic metal–organic frameworks and MOF-derived composites: Recent progress on electro-and photoelectrocatalytic applications. Coord Chem Rev.

[15] Yasin G, Ali S, Ibraheem S, Kumar A, Tabish M, Mushtaq MA, Ajmal S, Arif M, Khan MA, Saad A, Qiao L (2023). Simultaneously engineering the synergistic-effects and coordination-environment of dual-single-atomic iron/cobalt-sites as a bifunctional oxygen electrocatalyst for rechargeable zinc-air batteries. ACS Catal.

[16] Yasin G, Ibrahim S, Ajmal S, Ibraheem S, Ali S, Nadda AK, Zhang G, Kaur J, Maiyalagan T, Gupta RK, Kumar A (2022). Tailoring of electrocatalyst interactions at interfacial level to benchmark the oxygen reduction reaction. Coord Chem Rev.

[17] Baker R, Wilkinson DP, Zhang J (2009). Facile synthesis, spectroscopy and electrochemical activity of two substituted iron phthalocyanines as oxygen reduction catalysts in an acidic environment. Electrochim Acta.

[18] Baranton S, Coutanceau C, Roux C, Hahn F, Léger J-M (2005). Oxygen reduction reaction in acid medium at iron phthalocyanine dispersed on high surface area carbon substrate: tolerance to methanol, stability and kinetics. J Electroanal Chem.

[19] Zhang Z, Dou M, Ji J, Wang F (2017). Phthalocyanine tethered iron phthalocyanine on graphitized carbon black as superior electrocatalyst for oxygen reduction reaction. Nano Energy.

[20] Huang S, Chen K, Li T-T (2022). Porphyrin and phthalocyanine based covalent organic frameworks for electrocatalysis. Coord Chem Rev.

[21] Kuhl KP, Cave ER, Abram DN, Jaramillo TF (2012). New insights into the electrochemical reduction of carbon dioxide on metallic copper surfaces. Energy Environ Sci.

[22] Li CW, Kanan MW (2012). CO_2_ reduction at low overpotential on Cu electrodes resulting from the reduction of thick Cu_2_O films. J Am Chem Soc.

[23] Geng K, He T, Liu R, Dalapati S, Tan KT, Li Z, Tao S, Gong Y, Jiang Q, Jiang D (2020). Covalent organic frameworks: design, synthesis, and functions. Chem Rev.

[24] Kumar A, Das DK, Vashistha VK, Ibraheem S, Yasin G, Gautam S, Sharma V (2021). A novel CoN4-driven self-assembled molecular engineering for oxygen reduction reaction. Int J Hydrogen Energy.

[25] Kim D, Resasco J, Yu Y, Asiri AM, Yang P (2014). Synergistic geometric and electronic effects for electrochemical reduction of carbon dioxide using gold–copper bimetallic nanoparticles. Nat Commun.

[26] Zhao X, Pachfule P, Thomas A (2021). Covalent organic frameworks (COFs) for electrochemical applications. Chem Soc Rev.

[27] Lohse MS, Bein T (2018). Covalent organic frameworks: structures, synthesis, and applications. Adv Func Mater.

[28] Zhang S, Kang P, Meyer TJ (2014). Nanostructured tin catalysts for selective electrochemical reduction of carbon dioxide to formate. J Am Chem Soc.

[29] Lin S, Diercks CS, Zhang Y-B, Kornienko N, Nichols EM, Zhao Y, Paris AR, Kim D, Yang P, Yaghi OM (2015). Covalent organic frameworks comprising cobalt porphyrins for catalytic CO_2_ reduction in water. Science.

[30] Kumar A, Yasin G, Tabish M, Das DK, Ajmal S, Nadda AK, Zhang G, Maiyalagan T, Saad A, Gupta RK (2022). A catalyst-free preparation of conjugated poly iron-phthalocyanine and its superior oxygen reduction reaction activity. Chem Eng J.

[31] Xu S, Ding Y, Du J, Zhu Y, Liu G, Wen Z, Liu X, Shi Y, Gao H, Sun L (2022). Immobilization of iron phthalocyanine on pyridine-functionalized carbon nanotubes for efficient nitrogen reduction reaction. ACS Catal.

[32] Kumar A, Sun K, Duan X, Tian S, Sun X (2022). Construction of dual-atom fe via face-to-face assembly of molecular phthalocyanine for superior oxygen reduction reaction. Chem Mater.

[33] Wang Y, Li K, Cheng R, Xue Q, Wang F, Yang Z, Meng P, Jiang M, Zhang J, Fu C (2022). Enhanced electronic interaction between iron phthalocyanine and cobalt single atoms promoting oxygen reduction in alkaline and neutral aluminum-air batteries. Chem Eng J.

[34] Zhang Z, Wang W, Wang X, Zhang L, Cheng C, Liu X (2022). Ladder-type π-conjugated metallophthalocyanine covalent organic frameworks with boosted oxygen reduction reaction activity and durability for zinc-air batteries. Chem Eng J.

[35] Yang S, Li X, Tan T, Mao J, Xu Q, Liu M, Miao Q, Mei B, Qiao P, Gu S, Sun F, Ma J, Zeng G, Jiang Z (2022). A fully-conjugated covalent organic framework-derived carbon supporting ultra-close single atom sites for ORR. Appl Catal B.

[36] Ajmal S, Kumar A, Yasin G, Alam MM, Selvaraj M, Tabish M, Mushtaq MA, Gupta RK, Zhao W (2023). A microwave-assisted decoration of carbon nanotubes with Fe3O4 nanoparticles for efficient electrocatalytic oxygen reduction reaction. J Alloy Compd.

[37] Kumar A, Yasin G, Ajmal S, Ali S, Mushtaq MA, Makhlouf MM, Nguyen TA, Bocchetta P, Gupta RK, Ibraheem S (2022). Molecular MnN4-Complex immobilized on carbon black as efficient electrocatalyst for oxygen reduction reaction. Int J Hydrogen Energy.

[38] Vashistha VK, Kumar A (2020). Design and synthesis of MnN4 macrocyclic complex for efficient oxygen reduction reaction electrocatalysis. Inorg Chem Commun.

[39] Musa AB, Tabish M, Kumar A, Selvaraj M, Khan MA, Al-Shehri BM, Arif M, Mushtaq MA, Ibraheem S, Slimani Y (2023). Microenvironment engineering of Fe-single-atomic-site with nitrogen coordination anchored on carbon nanotubes for boosting oxygen electrocatalysis in alkaline and acidic media. Chem Eng J.

[40] Ma M, Kumar A, Wang D, Wang Y, Jia Y, Zhang Y, Zhang G, Yan Z, Sun X (2020). Boosting the bifunctional oxygen electrocatalytic performance of atomically dispersed Fe site via atomic Ni neighboring. Appl Catal B.

[41] Koçyiğit N, Özen ÜE, Özer M, Salih B, Özkaya AR, Bekaroğlu Ö (2017). Electrocatalytic activity of novel ball-type metallophthalocyanines with trifluoro methyl linkages in oxygen reduction reaction and application as zn-air battery cathode catalyst. Electrochim Acta.

[42] Chen K, Liu K, An P, Li H, Lin Y, Hu J, Jia C, Fu J, Li H, Liu HJNC (2020). Iron phthalocyanine with coordination induced electronic localization to boost oxygen reduction reaction. Science.

[43] Collman JP, Bencosme CS, Barnes CE, Miller BD (1983). Two new members of the dimeric. beta.-linked face-to-face porphyrin family: FTF4* and FTF3. J Am Chem Soc.

[44] Kumar A, Vashistha VK, Das DK (2021). Recent development on metal phthalocyanines based materials for energy conversion and storage applications. Coord Chem Rev.

[45] Kobayashi N, Nishiyama Y (1985). Catalytic electroreduction of molecular oxygen using iron or cobalt 4, 4', 4", 4'"-tetracarboxyphthalocyanine. J Phys Chem.

