# Supplementary material for: Fe-phthalocyanine derived highly conjugated 2D covalent organic framework as superior electrocatalyst for oxygen reduction reaction
Source: Discov Nano. 2023 Sep 4;18(1):109. doi: 10.1186/s11671-023-03890-w (PMC10477159; doi:10.1186/s11671-023-03890-w)
Supplement: Supplementary file 1 — Additional file 1. [file 11671_2023_3890_MOESM1_ESM.docx]

**Supporting Information**

# Fe-Phthalocyanine Derived Highly Conjugated 2D Covalent Organic Framework as Superior Electrocatalyst for Oxygen Reduction Reaction

Anuj Kumar ^a^, Mohd Ubaidullah ^b^, Bidhan Pandit ^f^, Ghulam Yasin ^c^ *, Ram K. Gupta ^d^ *, Guoxin Zhang ^e^ *

^a^ Nano-Technology Research Laboratory, Department of Chemistry, GLA University, Mathura, Uttar Pradesh-281406, India.

^b^ Department of Chemistry, College of Science, King Saud University, Riyadh-11451, Saudi Arabia.

^c^ Institute for Advanced Study, Shenzhen University, Shenzhen-518060, Guangdong, China.

^d^ Department of Chemistry, National Institute for Materials Advancement, Pittsburg State University, Pittsburg, KS-66762, USA.

^e^ Department of Electrical Engineering and Automation, Shandong University of Science and Technology, Qingdao, Shandong 266590, PR China

^f^ Department of Materials Science and Engineering and Chemical Engineering, Universidad Carlos III de Madrid, Avenida de la Universidad 30, 28911 Leganés, Madrid, Spain

**Corresponding Authors:**

Email: anuj.kumar@gla.ac.in (A. Kumar)

- 1. **Materials**

The ethyl alcohol (EtOH), and methanol (MeOH) were procured from TCI India. Sigma Aldrich supplied monomeric octa-amino-Fe-phthalocyanine and Nafion solution (0.5% wt. in alcohol). In the experiments, all chemicals and solvents were utilized exactly as they were procured.

- 1. **Physical Measurements**

Analytical tools like transmission electron microscopy (TEM), (FEI Tecnai G2 20), X-ray diffraction (XRD), (Shimadzu XRD-6000 with Cu K radiation (40kV, 30mA, = 1.5418 A)), and X-ray photoelectron spectroscopy (XPS), (PHI Quantera II XPS scanning microprobe), were utilized to evaluate the morphological, compositional and structural features of the prepared 2D FePc COF. A traditional three-electrode system including Ag/AgCl, a 5.0 mm glassy carbon (GC) disk as a working electrode, and a graphite rod as a counter electrode was utilized to evaluate the electrocatalytic performance of prepared 2D FePc COF.

- 1. **Electrochemical Measurements**

To perform the electrocatalytic ORR experiment, catalyst ink was prepared by taking 5 mg of 2D FePc-COF and 5 mg of carbon black in 10 μL of 5% Nafion and 0.5 mL of ethyl alcohol and sonicated for 30 min. Next, 5.0 μL of this catalyst ink got loaded on a GC electrode (0.250 mg cm^-2^ catalyst loading) and allowed to room temperature drying. Cyclic voltammograms (CVs) and linear sweep voltammograms (LSVs) were recorded in O_2_-soaked 0.1 M KOH electrolyte at 50 mV/s and 5 mV/s scan rates, respectively [1]. The electron transferred number (n) during ORR got computed by recording LSVs at various electrode rotating speeds between 625-2050 rpm and 5 mV/s, and employing the following equations[2].

$\frac{1}{J}=\frac{1}{J_{L}}+\frac{1}{J_{K}}=\frac{1}{B\omega^{\frac{1}{2}}}+\frac{1}{J_{K}}$ eq. (1)

$B=0.62nFC_{0}{D_{0}}^{\frac{2}{3}}\upsilon^{\frac{-1}{6}}$ eq. (2)

Where observed current density is represented by "*j*", "*j*_k_" shows kinetic current density as well as "*j*_L_" refers to limiting current density. The symbols ‘n and ω’ represent the no. of transferred ORR electrons and RDE rotation speed, respectively. C_0_ represents the strength of O_2_ in 0.1 mol L^-1^ KOH (1.2×10^-6^ mol cm^-3^), D_0_ is the diffusion coefficient of O_2_ in 0.1 mol L^-1^ KOH (1.9×10^-5^ cm^2^s^-1^), and F is the Faraday constant (96500 C). The conversion of measured potential against Ag/AgCl electrode to RHE electrode scale the Nernst equation was employed, as below.

$$E_{RHE}=E_{Ag/AgCl}+0.059pH+E_{Ag/AgCl}^{o}$$

- 1. **Theoretical Studies**

The Gaussian 09 software package was utilized for theoretical calculations. Functional B3LYP theory and 6-31G (d p) and SDD basis sets for C, H, N and Fe was exploited to optimize all geometries of OA-FePc and 2D FePc-COF systems [3]. Considering all the possible orientations, structural optimization was carried out to find the most stable configuration. Further, the adsorption energies of the oxygen-adducts of OA-FePc and 2D FePc-COF were computed using **eq. 1** [4].

ΔE_ads._ = E(Fe-N_4_/O-species) ‒ (E(Fe-N_4_) ‒ E(O_2_)) (**1)**

Where, E(Fe-N_4_/O-species), E(Fe-N_4_), and E(O-species) are the energies of O-species adsorbed on Fe-N_4_-adduct, Fe-N_4_ and O_2_, respectively.

**
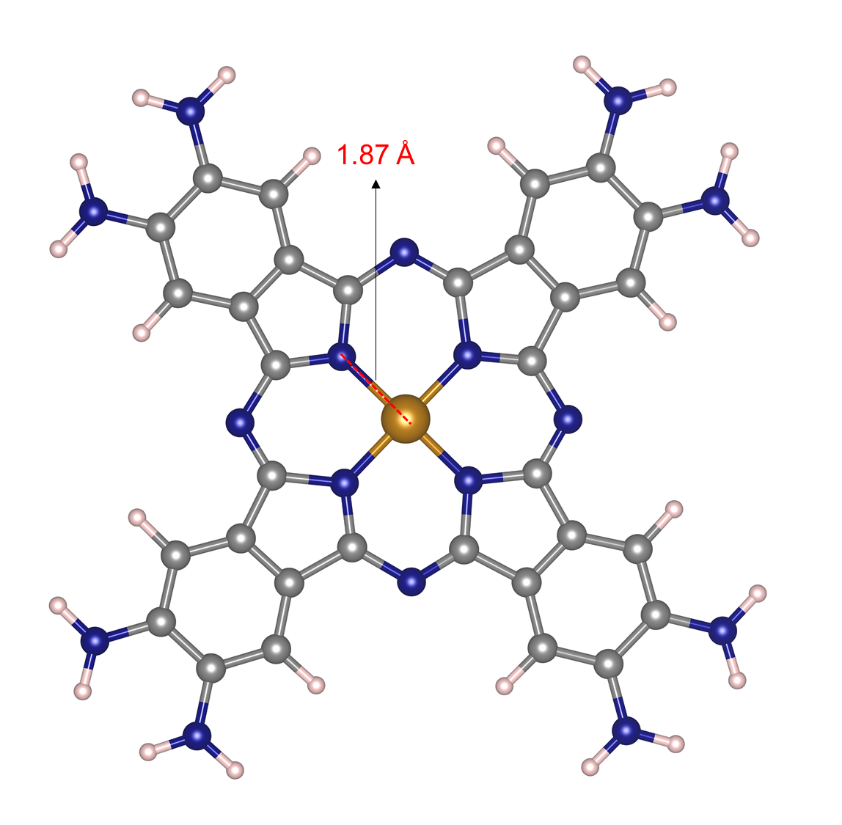
**

**Figure S1: Fe-N Bond length for optimized Monomeric FePc.**

**
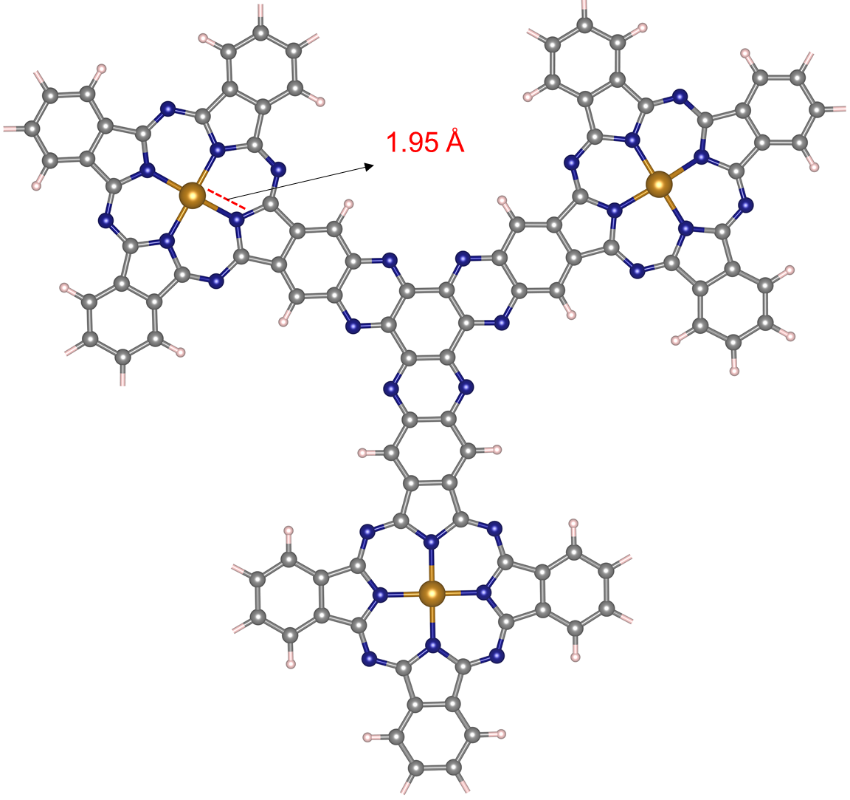
**

**Figure S2: Fe-N Bond length for optimized 2D FePc-COF.**

**
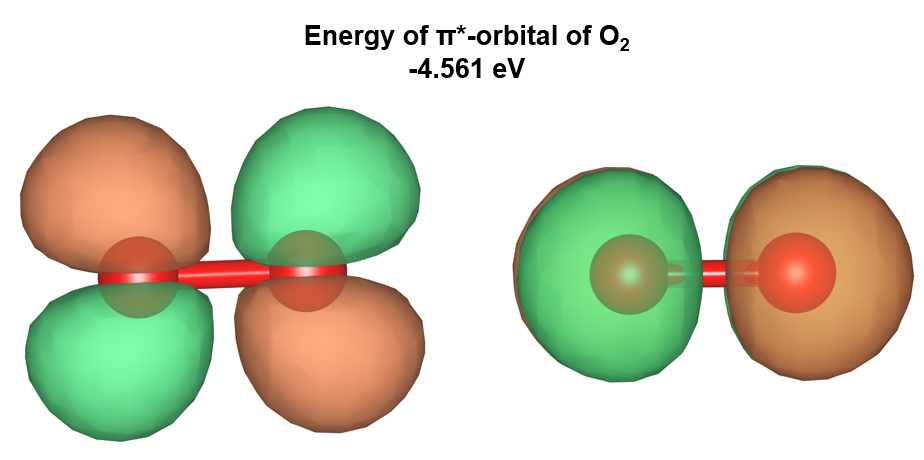
**

**Figure S3: Energy of optimized π*-orbital of O_2_.**


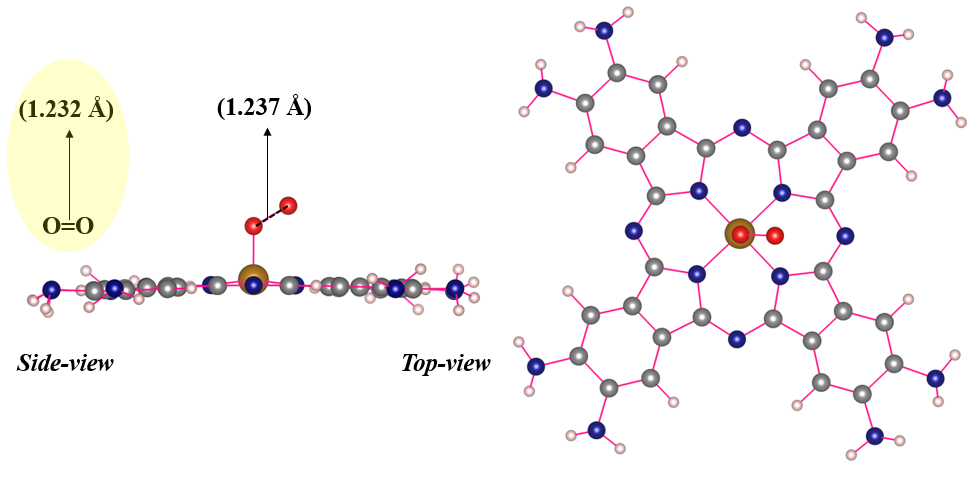


**Figure S4: O-O Bond length for OA-FePc-COF-O_2_ Adduct and free O_2._**


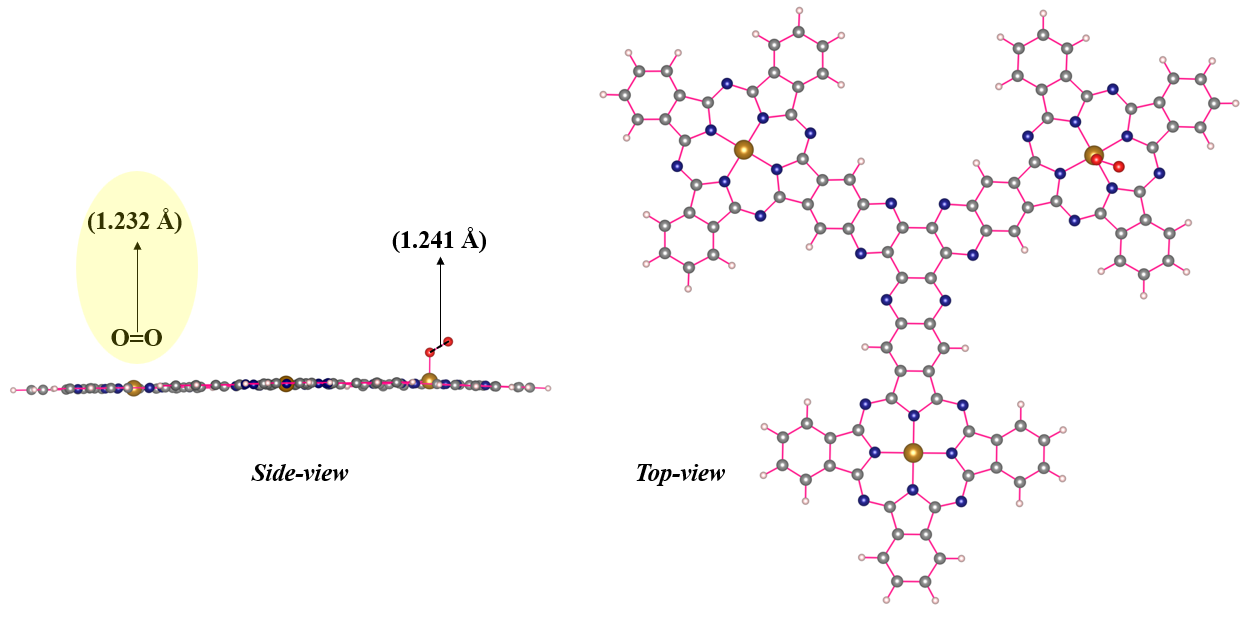


**Figure S5: O-O Bond length for 2D FePc-COF-O_2_ Adduct and free O_2_.**

**References**

[1] Z. Li, A. Kumar, N. Liu, M. Cheng, C. Zhao, X. Meng, H. Li, Y. Zhang, Z. Liu, G. Zhang, Oxygenated P/N co-doped carbon for efficient 2e− oxygen reduction to H 2 O 2, Journal of Materials Chemistry A, 10 (2022) 14355-14363.

[2] N. Liu, A. Kumar, Z. Li, Z. Liu, C. Zhao, X. Meng, Y. Wang, L. Yang, G. Zhang, Atomic Dual‐Site Ni/Co‐Decorated Carbon Nanofiber Paper for Efficient O2 Electrocatalysis and Flexible Zn‐Air Battery, ChemElectroChem, 9 (2022) e202200888.

[3] Z. Shi, J.J.T.J.o.P.C.C. Zhang, Density functional theory study of transitional metal macrocyclic complexes' dioxygen-binding abilities and their catalytic activities toward oxygen reduction reaction, 111 (2007) 7084-7090.

[4] S. Sun, N. Jiang, D.J.T.J.o.P.C.C. Xia, Density functional theory study of the oxygen reduction reaction on metalloporphyrins and metallophthalocyanines, 115 (2011) 9511-9517.
